# Reciprocal BLUP: A Predictability-Guided Multi-Omics Framework for Plant Phenotype Prediction

**DOI:** 10.3390/plants15010017

**Published:** 2025-12-20

**Authors:** Hayato Yoshioka, Gota Morota, Hiroyoshi Iwata

**Affiliations:** Graduate School of Agricultural and Life Sciences, University of Tokyo, Tokyo 113-8657, Japan; yoshioka@ut-biomet.org (H.Y.);

**Keywords:** genome, microbiome, metabolome, linear mixed model

## Abstract

Sustainable improvement of crop performance requires integrative approaches that link genomic variation to phenotypic expression through intermediate molecular pathways. Here, we present Reciprocal Best Linear Unbiased Prediction (Reciprocal BLUP), a predictability-guided multi-omics framework that quantifies the cross-layer relationships among the genome, metabolome, and microbiome to enhance phenotype prediction. Using a panel of 198 soybean accessions grown under well-watered and drought conditions, we first evaluated four direction-specific prediction models (genome → microbiome, genome → metabolome, metabolome → microbiome, and microbiome → metabolome) to estimate the predictability of individual omics features. We evaluated whether subsets of features with high cross-omics predictability improved phenotype prediction. These cross-layer models identify features that play physiologically meaningful roles within multi-omics systems, enabling the prioritization of variables that capture coherent biological signals enriched with phenotype-relevant information. Consequently, metabolome features were highly predictable from microbiome data, whereas microbiome predictability from metabolomic data was weaker and more environmentally dependent, revealing an asymmetric relationship between these layers. In the subsequent phenotype prediction analysis, the model incorporating predictability-based feature selection substantially outperformed models using randomly selected features and achieved prediction accuracies comparable to those of the full-feature model. Under drought conditions, the phenotype prediction models based on metabolomic or microbiomic kernels (MetBLUP or MicroBLUP) outperformed the genomic baseline (GBLUP) for several biomass-related traits, indicating that the environment-responsive omics layers captured phenotypic variations that were not explained by additive genetic effects. Our results highlight the hierarchical interactions among genomic, metabolic, and microbial systems, with the metabolome functioning as an integrative mediator linking the genotype, environment, and microbiome composition. The Reciprocal BLUP framework provides a biologically interpretable and practical approach for integrating multi-omics data, improving phenotype prediction, and guiding omics-based feature selection in plant breeding.

## 1. Introduction

Sustainable increases in agricultural productivity are essential to meet the demands of a growing global population. To address these challenges, modern plant breeding increasingly leverages genomic prediction approaches such as Genomic Best Linear Unbiased Prediction (GBLUP), which enables the accurate estimation of breeding values using genome-wide marker information [1]. Although genomic information alone can capture substantial genetic variance, complex traits are also shaped by intermediate molecular and ecological processes that cannot be fully explained by additive genetic effects [2,3].

Recent advances in multi-omics technologies have made it possible to profile diverse biological layers—genome [4], metabolome [5], and microbiome [6]—within the same individuals. These intermediate molecular layers provide unprecedented opportunities to unravel the complex causal relationships among biological systems, because they can act as mediators linking genotypes to phenotypes [7,8].

Among them, rhizosphere microorganisms play a pivotal role in plant growth and resilience by enhancing nutrient uptake, suppressing pathogens, and improving tolerance to abiotic stresses such as drought [9,10,11]. Therefore, understanding the structure and function of the rhizosphere microbiome is essential for deciphering the mechanisms of plant–microbe symbiosis and their contributions to stress adaptation [12].

The metabolome provides a complementary dimension by capturing the dynamic physiological state of plants under various environmental conditions. Because metabolite levels integrate genetic, environmental, and microbial influences, they can serve as sensitive biomarkers for stress responses and genotype-by-environment interactions [13,14].

Despite these advances, most existing frameworks treat all omics features equally, potentially diluting the biologically meaningful signals. While Christensen et al. [15] and Zhao et al. [16] focused on handling missing intermediate omics information within genetic evaluation frameworks, the distinct question of feature selection—identifying which omics variables are biologically relevant and predictive—has been less explored.

The need for feature selection arises partly from the characteristics of the intermediate omics layers. These layers are high-dimensional and often contain substantial redundancy and noise, making it challenging to determine the features that truly contribute to the transmission of biologically meaningful signals to the phenotype. This has motivated the development of biologically informed strategies to prioritize omics features.

In this study, we aimed to develop a reciprocal multi-omics framework to guide feature selection. We have quantified interactions and variance components across these interconnected layers in a plant–microbe system and evaluated whether cross-omics–informed feature subsets enhance phenotype prediction under contrasting environmental conditions.

To support this framework, we introduced the concept of cross-omics predictability—the extent to which one omics layer can be predicted from another—as a biologically grounded criterion for feature prioritization. This metric highlights the features that occupy functionally meaningful positions within the information flow from genotype to phenotype. Highly predictable features tend to represent coherent biological variations, including upstream–downstream regulatory relationships, stable genetic or ecological influences, and consistent responses to environmental perturbations. Therefore, prioritizing such features provides a principled strategy for dimensionality reduction that enriches phenotype-relevant signals while mitigating noise and potentially improves phenotype prediction accuracy.

In the following sections, we first describe the multi-omics datasets and experimental design employed in this study (Section 2.1). We then introduce our predictability-based integration framework, outline the implementation of inter-omics prediction and the identification of predictable features (Section 2.3), and describe how these selected omics features were used for phenotype prediction relative to genomic and random baselines (Section 2.5). The results of these analyses are presented in Section 3, followed by a discussion of their biological implications for understanding the plant–microbe–environment interactions and improving genomic prediction frameworks (Section 4).

## 2. Materials and Methods

### 2.1. Soybean Multi-Omics Data

We analyzed multi-omics datasets collected from a common panel of N=198 soybean accessions. The panel was drawn from the Global Soybean Minicore Collection [4]. The datasets included:

- Whole-genome genotypes (genome): a 198×425,858 matrix, where each column corresponds to a single nucleotide polymorphism marker in the soybean genome [4].
- Rhizosphere metabolome: a 198×265 matrix of metabolome features obtained from rhizosphere samples. Each column represents the normalized peak area of a distinct metabolome feature detected by tandem mass spectrometry [17].
- Rhizosphere microbiota profiles (microbiome): a 198×16,457 matrix generated by 16S rRNA gene amplicon sequencing of DNA extracted from rhizosphere samples. Each column represents the relative abundance of an amplicon sequence variant (ASV) [17].
- Plant phenotypes: a 198×9 matrix. These traits include biomass-related features such as shoot and leaf fresh/dry weights, growth stage, plant height, stem length, number of nodes, and number of branches.

All measurements were obtained from field trials conducted in 2019 under two watering conditions: a well-watered regime (control) and a water-limited regime (drought), enabling the assessment of environmental effects. Microbiome and metabolome datasets were identical to those described by Dang et al. [17] and were collected from the same field plots as the phenotypic measurements to ensure sample consistency across omics layers. Microbiome data were filtered to remove chloroplast and mitochondrial sequences, and all datasets were subsequently scaled prior to downstream analyses. The details of the data collection are described in Appendix A.

### 2.2. Theoretical Basis of Predictability as a Feature Selection Criterion

**Figure 1:**
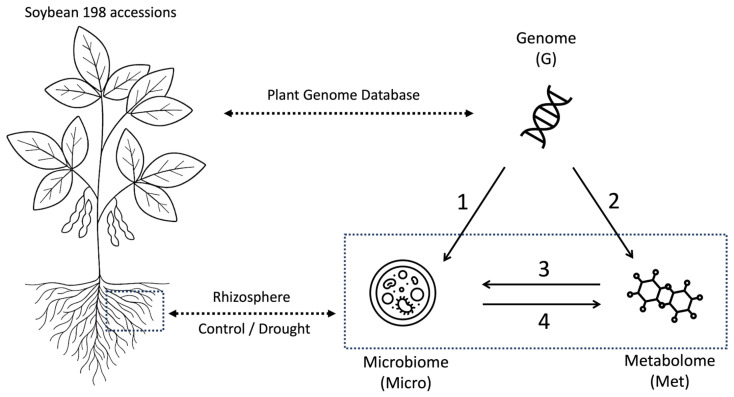
Conceptual overview of the modelling framework. We investigated how genomic (G), microbiome (Micro), and metabolome (Met) layers contribute to phenotype prediction across environments. Four direction-specific BLUP models were considered: (1) genome → microbiome, (2) genome → metabolome, (3) metabolome → microbiome, and (4) microbiome → metabolome. The microbiome and metabolome were sampled from the rhizosphere. The omics features were ranked by predictability in these directions, and the selected top features were evaluated in phenotype prediction.

In our framework, each microbiome feature or microbial taxon is treated as a quantitative trait, whose values are reconstructed from another omics layer (Figure 1). This allowed us to evaluate, for every feature, how strongly its variation followed patterns encoded in the genome, metabolome, or microbiome.

This study was conducted in two stages. First, we use predictability to rank the features and select subsets that represent meaningful cross-omics relationships (the “reciprocal” component of our framework). Second, the selected features were used to construct omics-derived kernels for phenotypic prediction. This two-stage design enabled us to evaluate whether focusing on the biologically coherent omics features—rather than using all variables—can improve phenotype prediction while providing interpretable insights into multi-layer interactions.

### 2.3. Predictability Analysis Among Omics Layers

We implemented a Best Linear Unbiased Predictor (BLUP)-based framework to integrate multiple omics layers using predictability-guided feature selection and phenotype prediction. Although BLUP was originally formulated to predict random effects based on expected pedigree relationships, its linear mixed-model framework was later extended to incorporate genome-wide markers (GBLUP). This extension enabled efficient analysis of high-dimensional genomic and other omics data [1,3].

This framework quantifies how omics features represent genetic and environmental signals, identifies stable high-predictability features across environments, and evaluates their contributions to the prediction of phenotypic values. The analysis focused on the interactions among the genome, microbiome, and metabolome layers under both control and drought conditions.

To characterize how different omics layers capture genetic and environmental information, we performed four direction-specific prediction analyses. We used BLUP models, each fitted with a single omics-derived relationship matrix (Figure 1):

**Model 1:** genome → microbiome
**Model 2:** genome → metabolome
**Model 3:** metabolome → microbiome
**Model 4:** microbiome → metabolome

Let $M=(m_{1},\ldots ,m_{q})$ denote the omics matrix to be predicted (e.g., metabolome or microbiome), and X the explanatory omics used as predictors. Here, when M represents the metabolome, $m_{j}$ denotes the vector of observed values for the *j*-th metabolite; whereas when M represents the microbiome, $m_{j}$ denotes vector of the observed abundances for the *j*-th ASV. For each response vector $m_{j}$, we fit a BLUP model to a single omics-derived relationship matrix:

(1) $$m_{j}=1\alpha_{j}+Za_{j}+\eta_{j},\;a_{j}∼N\;\left(0,\;\sigma_{a,j}^{2}\;K_{X}\right),\;\eta_{j}∼N\;\left(0,\;\sigma_{\eta ,j}^{2}I\right),$$

where $\alpha_{j}$ is the fixed-effect intercept for feature $m_{j}$, Z is the incidence matrix linking the samples to random effects, $a_{j}$ is the vector of omics-based random effects, and $\eta_{j}$ is the residual error term. The variance component $\sigma_{a,j}^{2}$ denotes the variance of the omics-based random effects, whereas $\sigma_{\eta ,j}^{2}$ represents the residual variance, which is specific to feature $m_{j}$.

The matrix $K_{X}$ represents the covariance structure of the omics-derived random effects. It is constructed from the explanatory omics data X as a linear relationship matrix:

(2) $$K_{X}=\frac{1}{q_{X}}XX^{T},$$

where $q_{X}$ is the number of explanatory features.

The predictive performance was evaluated using 5-fold cross-validation, and quantified using Pearson’s correlation (COR) between the observed and predicted values.

$$r_{j}=\mathrm{cor}\;\left(m_{j,\mathrm{test}},\;{\hat{m}}_{j,\mathrm{test}}\right).$$

### 2.4. Feature Selection Based on Predictability

The features were ranked according to their predictability score $r_{j}$, which quantifies how well each omics feature can be predicted from other data sources. Instead of applying a fixed correlation threshold, features were selected based on their relative predictability rankings. Specifically, we considered the top 100%, 50%, 25%, 12.5%, and 6.25% of features to correspond to progressively stricter levels of selection. This design allowed us to evaluate how stepwise feature reduction influences the prediction performance.

For each environment e∈{control,drought}, the top predictable features are defined as the top p% features ranked by $r_{j}^{\left(e\right)}$:

$$M_{\mathrm{top}}^{\left(e,p\right)}=\left\{\;j:r_{j}^{\left(e\right)}\;\mathrm{in}\;\mathrm{top}\;p\%\;\right\}.$$

where *p* denotes the proportion of retained features.

As a given omics layer may be predictable from multiple information sources, we combined the results across relevant models. For the metabolome, three feature-selection strategies were applied.

1. top:G—metabolites highly predictable from the genome;
2. top:Micro—metabolites highly predictable from the microbiome;
3. top:G+Micro—metabolites highly predictable from genome and microbiome.

Similarly, for the microbiome, features were selected using three analogous rules:

1. top:G—microbes predictable from the genome;
2. top:Met—microbes predictable from the metabolome;
3. top:G+Met—microbes predictable from genome and metabolome.

These top-feature sets $M_{\mathrm{top}}^{\left(e,p\right)}$ was used for downstream phenotype prediction analysis.

### 2.5. Phenotype Prediction Using Selected Omics Features

To evaluate the contribution of predictable omics features to phenotype prediction, we constructed linear kernels, which were used as sample-to-sample covariance structures (relationship matrices), from the features in each omics layer as follows:

$$K_{*}=\frac{1}{q_{*}}M_{*}M_{*}^{T},$$

where $M_{*}$ represents the feature matrix used to compute the kernel and $q_{*}$ is the number of features included. The following three feature sets were compared:

- Full omics ($M_{\mathrm{full}}^{\left(e\right)}$): all available features in the omics layer were used to construct the kernel, representing the baseline performance without feature filtering.
- Top omics ($M_{\mathrm{top}}^{\left(e,p\right)}$): only the top p% of features ranked by predictability in environment *e* were used. These represent the biologically and statistically predictable subsets identified through the predictability analysis.
- Random omics($M_{\mathrm{random}}^{\left(e,p,b\right)}$): a random subset of features with the same size as $M_{\mathrm{top}}^{\left(e,p\right)}$, generated for each iteration index *b* (b=1,…,10) to benchmark random selection against predictability-based selection.

These three settings enabled us to disentangle the effects of feature selection and model complexity on phenotype-prediction accuracy.

Phenotype prediction was performed using the BLUP model.

(3) $$y=1\mu +Zu+\varepsilon ,\;u∼N\;\left(0,\;\sigma_{u}^{2}K_{*}\right),\;\varepsilon ∼N\;\left(0,\;\sigma_{e}^{2}I\right).$$

Here, y denotes the vector of phenotypic values, μ is the fixed-effect intercept, and Z is the design matrix from samples to random effects. The vector u represents random effects with variance component $\sigma_{u}^{2}$, whereas ε denotes residual errors with variance $\sigma_{e}^{2}$.

The models based on metabolome and microbiome kernels are denoted as *MetBLUP* and *MicroBLUP*, respectively. The GBLUP model, using the additive genomic relationship matrix $K_{G}$, calculated according to Equation (2) is included as the baseline. The inclusion of GBLUP allows us to explicitly evaluate how additional omics layers improve conventional genotype–phenotype prediction.

The random feature setting serves as a conceptual benchmark that aligns with the scenarios considered in existing intermediate omics trait frameworks. In particular, Zhao et al. [16] examined prediction performance under conditions in which subsets of omics features were randomly missing. By contrast, our framework explicitly extends this scenario to situations in which feature availability can be actively controlled through feature selection. Comparison of this random omission baseline with predictability-guided feature selection allowed us to directly quantify the benefits of selecting informative features.

To account for the variability in random feature selection, the entire 5-fold cross-validation procedure (using five different seeds) was repeated ten times with a new random subset $M_{\mathrm{random}}^{\left(e,p,b\right)}$ generated at each iteration (b=1,…,10).

All the analyses were implemented in R  [18] using the RAINBOWR package [19]. Visualizations were produced using ggplot2 [20].

## 3. Results

### 3.1. Predictability Patterns Across Omics Layers and Environments

**Figure 2:**
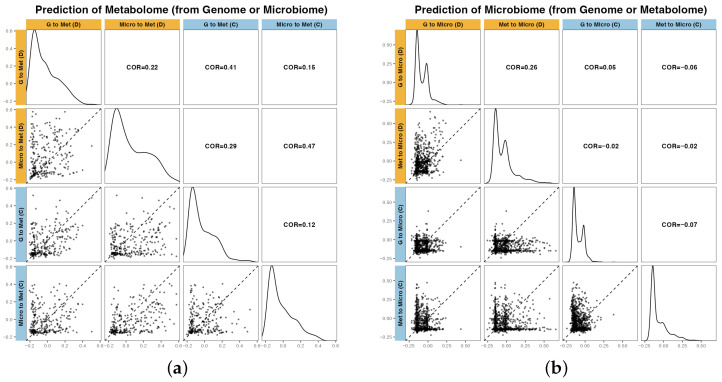
Prediction accuracy for each omics by method and environment. Left: (**a**) Metabolome; Right: (**b**) Microbiome. For the microbiome, Model 1 (G → Micro) and Model 3 (Met → Micro); for the metabolome, Model 2 (G → Met) and Model 4 (Micro → Met) are compared under Drought (D) and Control (C) conditions. Diagonal panels show the distributions of prediction accuracy of each omics feature for each setting; lower triangles display pairwise scatter plots of prediction accuracy for each omic feature (the dashed line indicates y=x); upper triangles report correlation coefficients between settings.

The pairwise prediction performances across omics layers are summarized in Figure 2. Microbiome prediction was more accurate when metabolome features were used as predictors (Model 3: Met → Micro) than when genome features were used as predictors (Model 1: G → Micro) under both control and drought conditions. Conversely, the prediction of the metabolome was more accurate when microbiome features served as predictors, particularly under drought conditions (Model 4: Micro → Met).

A similar pattern was observed for the number of features selected based on the predictability thresholds (Table 1). Model 1 (G → Micro) selected far fewer features with r>0.1, only 10/1193 (0.8%) under the control conditions and 35/893 (3.9%) under drought conditions. In contrast, Model 3 (Met → Micro) selected a larger number of metabolome features with high predictability (r>0.1), identifying 132/1193 (11.1%) under control conditions and 127/893 (14.2%) under drought conditions.

Similarly, Model 4 (Micro → Met) under drought conditions identified more predictable metabolome features than under control conditions, suggesting enhanced metabolic signaling to the microbial structure in water-limited environments.

### 3.2. Robust Metabolome Features Across Conditions and Models

**Figure 3:**
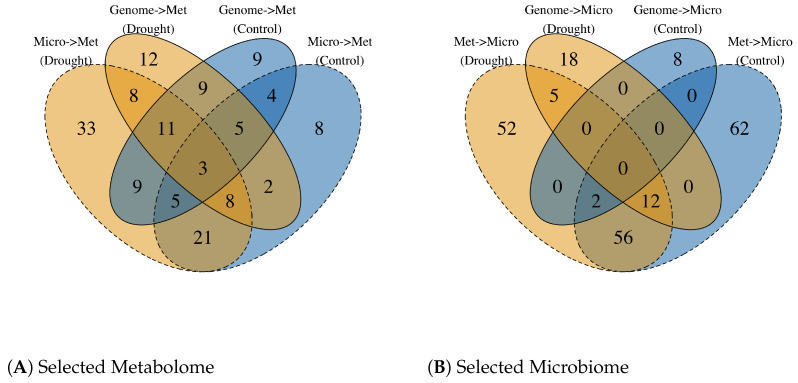
The selected microbiome and metabolome features under each condition. Drought: yellow; Control: blue. Identified using Pearson correlation (r>0.1). (**A**) For the prediction of the metabolome, genomic or microbiome features were used as inputs. (**B**) For the prediction of the microbiome, genomic or metabolome features were used as inputs.

A Venn diagram summarizing the features exceeding r>0.1 across the four models and the two environments (Figure 3) revealed a set of robust metabolome features that were consistently predictable, regardless of the model direction or environment. Three metabolites—glutamic acid, daidzein, and genistin—were consistently selected across all conditions. These compounds have been implicated in root exudation, plant–microbe interactions, and stress responses in the rhizosphere, underscoring their biological relevance [21,22,23].

In contrast, no single bacterial taxon was consistently predictable across the environments or models. Microbiome feature selection exhibited pronounced environment- and genome-dependent variation.

### 3.3. Phenotype Prediction Using Predictability-Selected Omics Features

#### 3.3.1. MetBLUP: Phenotype Prediction by Selected Metabolome

**Figure 4:**
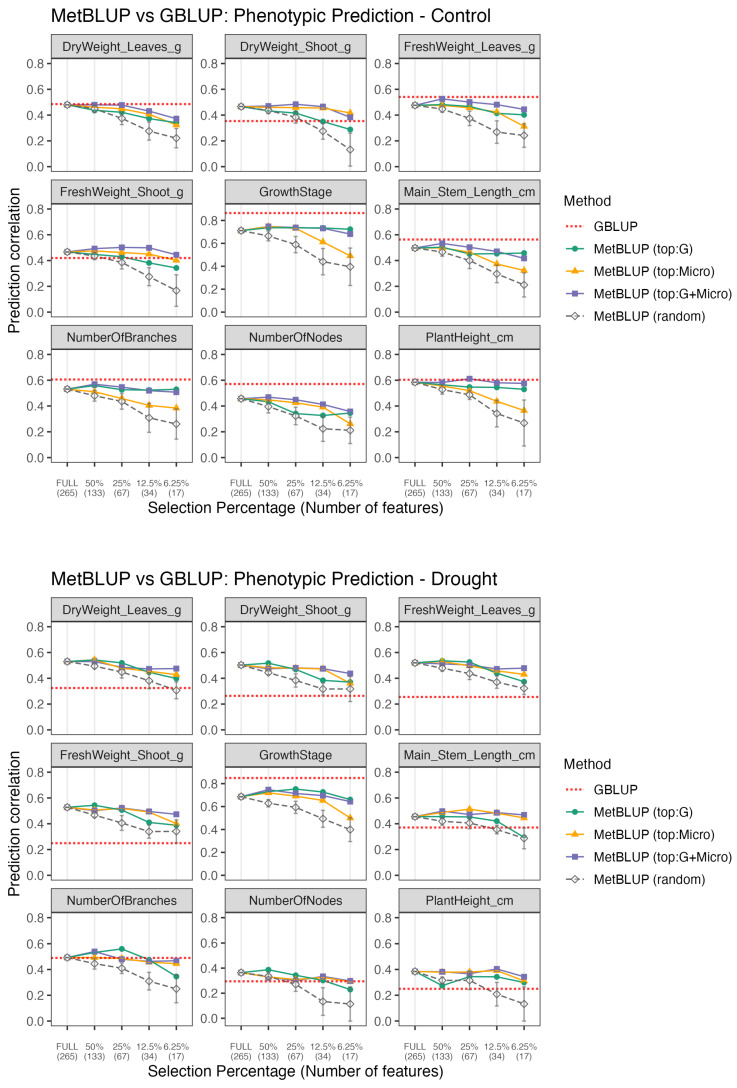
Phenotype prediction via MetBLUP across selection thresholds. The x-axis shows selection percentages, with the number of selected features at each threshold in parentheses. Solid lines represent models based on metabolome features selected by different methods: “top:G” (features selected using genomic information), “top:Micro” (features selected using microbiome information), and “top:G+Micro” (features jointly selected using both genomic and microbiome data). Dashed gray lines correspond to random feature selections matched by feature count. The dotted red horizontal lines indicate the prediction accuracy obtained by GBLUP, used here as a genomic baseline. For each point, the marker indicates the mean prediction correlation from 5-fold CV with five different seeds. For the random model, this procedure was repeated 10 times; the error bars represent the standard deviation across iterations. For the other models (top:G, top:Micro, top:G+Micro), the kernel is identical across iterations; accordingly, no error bars are shown.

Next, we assessed whether predictability-based feature selection enhanced phenotype prediction. For metabolome-based models (MetBLUP; Figure 4), the models based on the most predictable features consistently outperformed those built from randomly selected subsets of equal size, and their predictive accuracy was often comparable to or greater than that of the full-feature models.

Overall, reducing the feature set from 265 to 34 features (25%) resulted in only a negligible reduction in prediction accuracy compared to using the full 265-feature set.

At each feature count level, the predictability-based subsets consistently outperformed the random subsets matched by the feature count. Notably, the performance difference between the selected and random subsets increased as the number of features decreased.

For most phenotypes, MetBLUP outperformed the genomic baseline (GBLUP) under drought conditions and achieved comparable accuracy under control conditions.

When comparing the three feature-selection rules (top:G, top:Micro, and top:G+Micro), distinct trends emerged depending on the trait. The top:Micro performed best for traits with lower genomic predictability (e.g., shoot dry weight), whereas top:G yielded the highest accuracy for the traits that were already well predicted by GBLUP (e.g., growth stage). The top:G+Micro rule provided complementary information, resulting in the most stable and robust performance across traits.

A similar trend was observed when comparing the two conditions, where GBLUP predictability was generally lower under drought conditions, whereas MetBLUP based on top:G+Micro features, achieved superior performance for major biomass traits (such as leaf dry weight), highlighting the influence of environmental factors mediated by metabolomic contributions.

#### 3.3.2. MicroBLUP: Phenotype Prediction by Selected Microbiome

**Figure 5:**
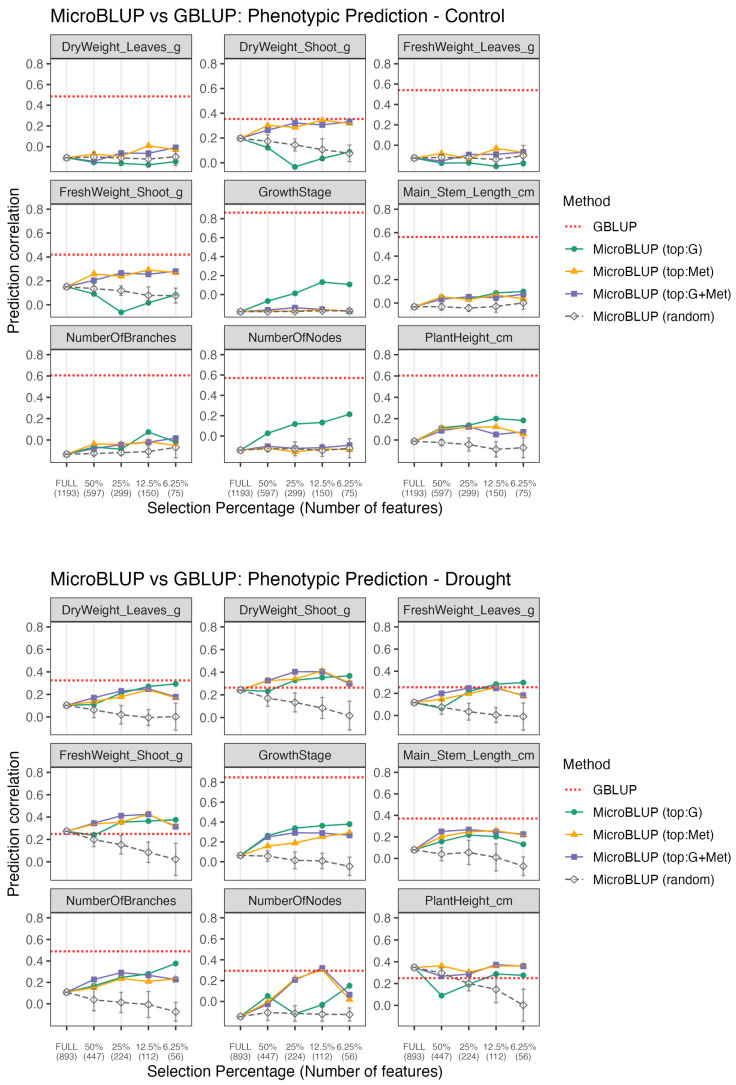
Phenotype prediction via MicroBLUP across selection thresholds. The x-axis shows selection percentages, with the number of selected features at each threshold in parentheses. Solid lines represent models based on microbiome features selected by different methods: “top:G” (features selected using genomic information), “top:Met” (features selected using metabolomic information), and “top:G+Met” (features jointly selected using both genomic and metabolomic data). Dashed gray lines correspond to random feature selections matched by feature count. The dotted red horizontal line indicates the prediction accuracy obtained by GBLUP, used here as a genomic baseline. For each point, the marker indicates the mean prediction correlation from 5-fold CV with 5 different seeds. For the random model, this procedure was repeated 10 times; the error bars represent the standard deviation across iterations. For the other models (top:G, top:Met, top:G+Met), the kernel is identical across iterations; accordingly, no error bars are shown.

For microbiome-based models (MicroBLUP; Figure 5), models using the top predictable features also significantly outperformed random subsets of equal size. Under drought conditions, models using only the top 6.25% of microbiome features achieved predictive abilities comparable to those of or even exceeded those using all features, and were on par with the GBLUP baseline for most biomass-related phenotypes, such as leaf dry weight.

However, under the control conditions, the predictive ability was generally lower than that of GBLUP. Notably, for major biomass-related traits, such as shoot weight and leaf weight under the control conditions, top:Met-based feature selection outperformed top:G. This suggests that microbiome features predictable from metabolomic data better capture the relevant biological variations in these traits. In contrast, under the drought conditions, the difference between the selection rules was smaller. In several cases where the microbiome was strongly filtered (6.25%), top:G achieved a slightly higher accuracy than top:Met, indicating a shift in the dominant sources of microbiome predictability across environments.

## 4. Discussion

### 4.1. Predictive Asymmetry Between Omics Layers

Our results revealed a clear asymmetry in the predictive power of the metabolome and microbiome layers. The metabolome was highly predictable from microbiome data, whereas the reverse direction showed weaker and more environment-sensitive patterns (Figure 2). Moreover, the selected microbiome features were specific to each drought and control treatment (Figure 3). This finding is consistent with the notion that metabolite pools primarily represent the downstream outputs of plant physiology and that these metabolic outputs shape the microbial community structure. Consequently, the metabolome provides relatively stable biological signals, whereas the microbiome is more sensitive to environmental fluctuations.

### 4.2. Genetic Versus Environmental Contributions to Phenotype Prediction

The predictability-based feature selection approach provides a unique perspective on how genetic and environmental factors shape phenotypic variation in different omics layers. Traits with high genomic predictability tended to show better phenotypic prediction accuracy when the top:G-selected features were used, particularly in metabolome-based models (MetBLUP) (Figure 4). This suggests that, for highly heritable traits, the metabolome captures genotype-associated physiological variations. In contrast, traits with lower genomic predictability or stronger environmental influences benefited more from the top:Micro feature sets, indicating that metabolomic data also capture environmentally responsive biochemical or physiological processes that are not directly encoded in the genome. In the present study, biomass-related traits were relatively sensitive to microbiome and metabolome features, whereas traits such as growth stage, number of branches, and plant height were more strongly driven by genetic variation.

### 4.3. Environmental Modulation of Omics–Phenotype Relationships

Under drought stress, genomic predictability (GBLUP accuracy) was generally reduced, whereas the phenotype prediction performance of MetBLUP and MicroBLUP models improved (Figure 4 and Figure 5). This shift implies that environmental perturbations enhance the relevance of metabolomic and microbial responses to stress, leading to a greater contribution from environment-associated variations. Thus, whereas genetic control dominates under optimal conditions, environmental modulation of the omic layer becomes a major driver of phenotypic variation under stress.

### 4.4. Hierarchical Relationships Among Genome, Metabolome, and Microbiome

The relationships between the three omics layers imply a hierarchical association structure. The genome and metabolome showed strong associations, consistent with a pattern in which genomic variation is related to metabolic states and, in turn, to phenotypic variation in biomass-related traits (Figure 4). In contrast, the microbiome exhibits weaker direct associations with the genome, as reflected by the limited ability of genome-based models to predict microbial composition compared with metabolome-based predictions (Figure 3). We also observed that the top:G-based MicroBLUP model showed limited predictive ability for phenotypic traits, particularly under control conditions (Figure 5). Furthermore, microbial composition was more strongly associated with metabolomic data than with genomic data. Given that metabolomic features were comparably predictable from both the genome and microbiome (Figure 3). These patterns suggest a possible hierarchical association structure in which genomic variation is linked to metabolomic profiles, which are further associated with variations in rhizosphere microbial communities.

### 4.5. Biomarker Selection

Our reciprocal framework provides a practical approach for prioritizing omics features based on their predictability and leveraging them for phenotype prediction. In the metabolome, the filtered feature sets (25%) performed comparably to the full dataset in the MetBLUP framework, whereas in the microbiome, an even smaller subset of features (6.25%) outperformed the full dataset in the MicroBLUP framework (Figure 4 and Figure 5).

Several previous studies have investigated the development and improvement of phenotype prediction models using omics data, including metabolomic and microbiome information [3,5,24]. These past studies primarily aimed to enhance predictive performance by incorporating additional omics layers or developing novel modeling strategies.

In parallel, intermediate omics trait models have been proposed to explicitly model the dependencies and information flows between different omics layers [15,16]. While these frameworks provide important insights into cross-omics relationships, they are not designed to perform feature selection or optimize predictive performance.

In contrast, the key novelty of the present study lies in the systematic evaluation of feature selection based on cross-omics predictability with the explicit goal of improving phenotype prediction. Our results have demonstrated that predictability-guided feature prioritization can substantially reduce the dimensionality of omics data while maintaining or even improving predictive accuracy, highlighting its practical utility for multi-omics genomic prediction.

### 4.6. Practical Deployment Considerations

Although multi-omics integration improves prediction, its deployment in breeding is limited by the high cost of metabolomic and microbiome profiling and the lack of temporal measurements. Our framework helps to mitigate these constraints by identifying compact, biologically interpretable subsets of features. For the metabolome in particular, reducing the number of features has practical benefits, as targeted assays can become substantially less costly when only a small set of compounds needs to be measured. Thus, even in resource-limited breeding programs, focusing on a small set of informative biomarkers provides a feasible and cost-effective alternative for full multi-omics profiling. It may be feasible to extend these strategies to other crops using this framework.

### 4.7. Limitations

This study had several limitations that warrant consideration. First, the analyses were conducted at a single location during the growing season. In future work, multi-environment and multi-year trials will be essential to assess the generalizability of the framework and capture temporal dynamics, including potential genotype-by-environment (G × E) interactions. Second, the sample size (N = 198) may have limited the ability to detect subtle associations, particularly in high-dimensional settings. Third, the current implementation relies on linear BLUP-based models for predictability estimation, and nonlinear models may further improve the capture of complex interactions between omics layers. Further experimental validation is required to establish the direct biological functions of the identified features.

## 5. Conclusions

Our findings have highlighted the fact that integrating predictability-based feature selection enables the disentanglement of genetic and environmental contributions to complex traits. The proposed framework clarifies the distinct yet interconnected roles of the genome, metabolome, and microbiome. Moreover, our approach enabled the selection of biologically meaningful features and enhanced the performance of phenotype prediction models. Such an understanding is critical for improving multi-omics prediction models and designing strategies to exploit both genetic and environmental variations for broader crop improvement.

## Figures and Tables

**Table 1:** Number of selected features. The correlation (r) threshold for each direction-specific prediction model. The Full column shows the total number of available features without correlation filtering.

Model	Environment	r>0.3	r>0.2	r>0.1	r>0	Full
Model 1: G → Micro	Control	1	3	10	91	1193
	Drought	1	4	35	105	893
Model 2: G → Met	Control	8	16	55	95	265
	Drought	7	28	58	91	265
Model 3: Met → Micro	Control	15	55	132	263	1193
	Drought	38	68	127	233	893
Model 4: Micro → Met	Control	11	22	56	95	265
	Drought	39	72	98	131	265

## Data Availability

All source codes and data are available from the repository in GitHub: https://github.com/Yoska393/ReciprocalBLUP (accessed on 20 November 2025).

## Appendix A. Multi-Omics Data Collection

### Appendix A.1. Field Experiment

A total of 198 diverse soybean accessions from the NARO Genebank (https://www.gene.affrc.go.jp; accessed on 20 November 2025), representing the Global Soybean Minicore Collection [4,25], were cultivated at the Arid Land Research Center of Tottori University in 2019. Field management followed that of Dang et al. [17], Toda et al. [26], Sakurai et al. [27]. Plants were grown in sandy soil under two watering regimes: well-watered control and drought (non-irrigated) conditions. Fertilizers were applied prior to sowing and irrigation was controlled using buried drip tubes under white mulch sheets. Biomass-related phenotypic measurements and rhizosphere root samples were collected from the same plots at the beginning of September to ensure data consistency across omics layers. The root samples were immediately frozen at −80 °C for metabolome and microbiome analyses. The soybean genome sequences are available from the DDBJ Sequence Read Archive (BioProject accession: PRJDB7281).

### Appendix A.2. Phenotypic Traits

Nine biomass-related traits were measured: the dry and fresh weights of shoots and leaves, growth stage, plant height, stem length, number of nodes, and number of branches.

### Appendix A.3. Metabolome Analysis

Root metabolomic profiling was conducted following the methods described by Sawada et al. [28] and Uchida et al. [29]. Freeze-dried and powdered root samples were extracted using 80% methanol containing internal standards, analyzed using LC–QqQ–MS (LCMS-8050, Shimadzu, Kyoto, Japan), and quantified in the multiple reaction monitoring (MRM) mode. The peak areas were processed using MRMPROBS [30] and normalized against internal standards. A processed metabolomic dataset was obtained from the RIKEN DropMet database (ID: DM0071).

### Appendix A.4. Microbiome Analysis

The root-associated and rhizosphere bacterial communities were characterized using 16S rRNA gene sequencing. Genomic DNA was extracted as described previously Kumaishi et al. [31] and Ichihashi et al. [32]. The V4 region of the 16S rRNA gene was amplified using a two-step PCR protocol with PNA blocking primers [33,34]. Amplicons were sequenced on an Illumina MiSeq platform (2 × 300 bp). Raw reads were processed using QIIME2 (ver. 2020.6.0), using Cutadapt and DADA2 [35] for quality filtering, denoising, and chimeric removal. Taxonomic assignments were performed using the SILVA database (ver. 138) [36,37], and non-bacterial sequences were excluded. Rarefaction was performed (n=3000), resulting in 1642 ASVs. Additional filtering was performed separately for each condition. The final dimensions of the data were 179×1193 for the control condition and 182×893 for the drought conditions, respectively. Raw sequencing data are available from DDBJ (BioProject accession: PRJDB37881).

## Appendix B. BLUP for Multi-Omics Data

The predictive performance of BLUP-based methods has been demonstrated in omics and multi-omics studies [1,3,5]. The n≪p setting is common in quantitative genetics and omics research, where genomic datasets contain millions of SNPs. GBLUP is mathematically equivalent to ridge regression and therefore mitigates overfitting by implicitly using an L2 penalty [38].

Moreover, GBLUP relies on covariance structures rather than explicit feature-wise parameter estimation, making it particularly efficient in high-dimensional settings where the number of features greatly exceeds the sample size.

To capture nonlinear relationships, kernel extensions of BLUP, such as Gaussian kernels, can be introduced. However, previous studies have reported that the predictive performance of nonlinear kernels is often comparable to that of linear kernels in the context of genomic prediction [14].

In terms of prediction accuracy for typical phenotypic prediction tasks, BLUP and random forest (RF)—a widely used and robust nonlinear machine learning method—have been reported to achieve comparable performance [39,40]. In specific scenarios where strong nonlinear effects are expected, nonlinear models such as RF may be effective [24].

Across a wide range of practical applications, BLUP provides competitive predictive performance while offering advantages in terms of model interpretability, variance component decomposition, and also stable estimation in high-dimensional omics settings.
